# Electrical Stimulation of the Primate Lateral Habenula Suppresses Saccadic Eye Movement through a Learning Mechanism

**DOI:** 10.1371/journal.pone.0026701

**Published:** 2011-10-24

**Authors:** Masayuki Matsumoto, Okihide Hikosaka

**Affiliations:** 1 Laboratory of Sensorimotor Research, National Eye Institute, National Institutes of Health, Bethesda, Maryland, United States of America; 2 Primate Research Institute, Kyoto University, Inuyama, Aichi, Japan; University of New South Wales, Australia

## Abstract

The lateral habenula (LHb) is a brain structure which represents negative motivational value. Neurons in the LHb are excited by unpleasant events such as reward omission and aversive stimuli, and transmit these signals to midbrain dopamine neurons which are involved in learning and motivation. However, it remains unclear whether these phasic changes in LHb neuronal activity actually influence animal behavior. To answer this question, we artificially activated the LHb by electrical stimulation while monkeys were performing a visually guided saccade task. In one block of trials, saccades to one fixed direction (e.g., right direction) were followed by electrical stimulation of the LHb while saccades to the other direction (e.g., left direction) were not. The direction-stimulation contingency was reversed in the next block. We found that the post-saccadic stimulation of the LHb increased the latencies of saccades in subsequent trials. Notably, the increase of the latency occurred gradually as the saccade was repeatedly followed by the stimulation, suggesting that the effect of the post-saccadic stimulation was accumulated across trials. LHb stimulation starting before saccades, on the other hand, had no effect on saccade latency. Together with previous studies showing LHb activation by reward omission and aversive stimuli, the present stimulation experiment suggests that LHb activity contributes to learning to suppress actions which lead to unpleasant events.

## Introduction

The lateral habenula (LHb) is a structure which belongs to the habenular complex in the epithalamus. It has been described as an important relay station carrying information from the forebrain limbic system down to midbrain structures [1], [2]. Its efferents reach GABAergic neurons in the rostromedial tegmental nucleus, which in turn projects to the ventral tegmental area and substantia nigra pars compacta containing dopamine neurons and possibly to the dorsal raphe nucleus containing serotonin neurons [3], [4], [5], [6], [7]. Thus, the LHb is in a good position to regulate the dopaminergic and serotonergic systems which influence emotion and motivation [8], [9]. Consistent with this view, the habenula has been implicated in many functions such as anxiety [10], stress [11], pain [12], maternal behavior [13], attention [14], error monitoring [15] and learning [16], [17].

The understanding of LHb function has been advanced by recent studies which determined how LHb neurons are activated during animal behavior. These studies found that LHb neurons are inhibited by pleasant events such as rewards and their predictors and excited by unpleasant events such as reward omission, aversive stimuli and their predictors, suggesting negative value coding by these neurons [18]. Then, the negative value signals are transmitted to midbrain dopamine neurons by inhibiting them [19]. Since theoretical and experimental studies have suggested the involvement of dopamine neurons in reward-seeking and punishment-avoidance learning [20], [21], [22], [23], [24], it is possible that the LHb signals also contribute to the learning by influencing dopamine neuron activity. However, it remains unclear whether these phasic LHb signals actually influence animal behaviors, and if so, how.

Using a visually guided saccade task in monkeys, we previously found that LHb neurons were excited when the monkeys were required to make a saccade which was not associated with reward [19]. Saccade latencies for those no-rewarded saccades were longer compared with rewarded saccades. These findings suggest that negative events (e.g., no reward) can suppress saccadic eye movements and that LHb activity might be a teaching signal to cause this suppression. To test this hypothesis, here we examined the effect of electrical stimulation of the LHb on saccadic eye movements using a similar experimental design. Instead of omitting reward after saccades, we now simply delivered electrical stimulation of the LHb. This experimental design enabled us to test whether LHb stimulation could act as a substitute for reward omission in suppressing animal behaviors. In fact, we found that the latency of the saccade that was followed by LHb stimulation increased gradually as the stimulation was repeated.

## Materials and Methods

### Animals

Two adult rhesus monkeys (Macaca mulatta; monkey E, male, 8.5 kg; monkey D, male, 11.0 kg) were used for the experiments. All procedures for animal care and experimentation were approved by the Animal Care and Use Committee of the National Eye Institute (Animal Study Proposal Number  =  05–540) and complied with the Public Health Service Policy on the humane care and use of laboratory animals. All efforts were made to minimize suffering in accordance with the recommendations of the “The use of non-human primates in research”. For example, the monkeys were kept in individual primate cages in an air-conditioned room where food was always available. Their health condition, such as body weight and appetite, was checked daily. Supplementary water and fruit were provided daily.

### Surgery

A plastic head holder and plastic recording chamber were fixed to the skull under general anesthesia and sterile surgical conditions. The recording chamber was placed over the midline of the parietal cortex and was aimed at the LHb. Two search coils were surgically placed under the conjunctiva of the eyes for measurement of eye position. The head holder, the recording chamber and the eye coil connectors were all embedded in dental acrylic that covered the top of the skull and were connected to the skull by acrylic screws.

### Electrophysiology

Electrical stimulations were performed using tungsten electrodes (Frederick Haer Company, Bowdinham, ME) that were advanced by an oil-driven micro-manipulator (MO-97A, Narishige, Japan). The electrical stimulation sites were determined using a grid system which allowed electrode penetrations with 1 mm spacing. The electrode was introduced into the brain through a stainless steel guide tube which was inserted into one of the grid holes and then into the brain via the dura. For finer mapping, we also used a complementary grid which allowed electrode penetrations between the holes of the original grid, allowing penetrations with 0.5 mm spacing.

### Behavioral paradigm

A trial started when a small fixation spot appeared on the screen. After the monkeys maintained fixation on the spot for 1200 ms, the fixation spot disappeared and a peripheral target appeared at either right or left, 15° from the fixation spot. The monkeys were required to make a saccade to the target within 1000 ms. Correct and incorrect saccades were signaled by tone and beep stimuli 200 ms after the saccades. The correct saccades were followed by a fixed amount of liquid reward in half of the trials in both directions and were followed by no reward in the other half of trials. The rewarded and unrewarded trials were determined randomly. In the rewarded trials, reward delivery started simultaneously with the onset of the tone stimulus. In one block of 36 trials, saccades to one fixed direction were followed by unilateral electrical stimulation of the LHb (monkey E, 20 or 40 µA, 0.4 ms pulse, 300 Hz, 800 ms duration; monkey D, 40 µA, 0.4 ms pulse, 300 Hz, 1500 ms duration) while saccades to the other direction were not. The stimulation parameters were determined in a preliminary experiment in which we found a significant stimulation effect on saccadic latency. The stimulation onset was synchronized with the time when the delivery of liquid reward would be started. The direction-stimulation contingency was reversed in the next block with no external instruction. The reversal was repeated 7 times for each experiment.

As a control, we also stimulated the LHb during the preparation and execution of saccade in one monkey (monkey E). The task procedure and stimulation parameters were the same as the original visually guided saccade task except the timing of electrical stimulation and the timing of reward delivery. The electrical stimulation started simultaneously with the target onset and continued for 800 ms. To avoid the overlap between the electrical stimulation and reward- or postreward-period, reward delivery was delayed by 700 ms compared with the original task.

The data from monkey E and monkey D obtained using the different stimulation parameters were pooled for subsequent population analyses. For statistical comparisons, we applied the Wilcoxon rank-sum test to non-pair comparisons and the Wilcoxon signed-rank test to pair comparisons.

### Electrical stimulation sites

Identification of LHb stimulation sites was done using the same procedure as reported previously [18], [19]. We estimated the position of the LHb by obtaining MRIs (4.7 T, Bruker, Germany). We then recorded from neurons in and around the estimated LHb, and found that the firing patterns and spike shapes within the estimated LHb were distinctly different from neurons in the surrounding thalamic area, the mediodorsal nucleus of the thalamus (MD). Furthermore, most of the presumed LHb neurons, but none of the presumed MD neurons, were sensitive to reward outcome. Importantly, the characteristics of firing and the relation to reward outcome were distinctly different between the estimated LHb and the surrounding areas, even when they were separated only by 0.5 mm or 1.0 mm. We therefore regarded the estimated LHb as the actual LHb.

In order to examine the effect of electrical stimulation of the LHb, we first recorded single- or multi-unit activity in the LHb that was modulated by reward outcome in the visually guided saccade task, and then used the recording electrode for electrical stimulation. Most of the recorded single- and multi-unit activities showed an inhibitory response to reward and/or excitatory response to no-reward.

We also stimulated the MD. The stimulation sites were 1 to 2 mm away from the LHb laterally. Before stimulation experiment, we also recorded single- or multi-unit activities in the MD and confirmed the physiological properties.

## Results

**Figure 1:**
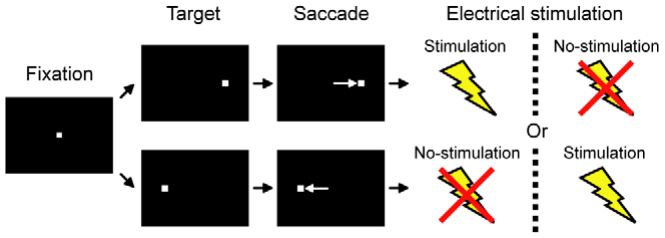
The visually guided saccade task. A trial started when a small fixation spot appeared on the screen. After the monkeys maintained fixation on the spot for 1200 ms, the fixation spot disappeared and a peripheral target appeared at either right or left. The monkeys were required to make a saccade to the target within 1000 ms. In one block of 36 trials, saccades to one fixed direction were followed by electrical stimulation of the LHb while saccades to the other direction were not. The direction-stimulation contingency was reversed in the next block.

We performed 35 experiments in which the LHb was electrically stimulated in two monkeys. The behavioral paradigm is diagramed in Fig. 1. A saccade target was presented randomly on the left or right and the monkey had to make a saccade to it immediately, after which a fixed amount of liquid reward was delivered with 50% probability. In one block of 36 trials, saccades to one fixed direction were followed by electrical stimulation of the LHb while saccades to the other direction were not. The direction-stimulation contingency was reversed in the next block. We compared saccade latency between the stimulation condition (that is, saccades followed by LHb stimulation) and the no-stimulation condition (that is, saccades not followed by stimulation) for each saccade direction.

### Effects of electrical stimulation of the LHb on saccade latency

**Figure 2:**
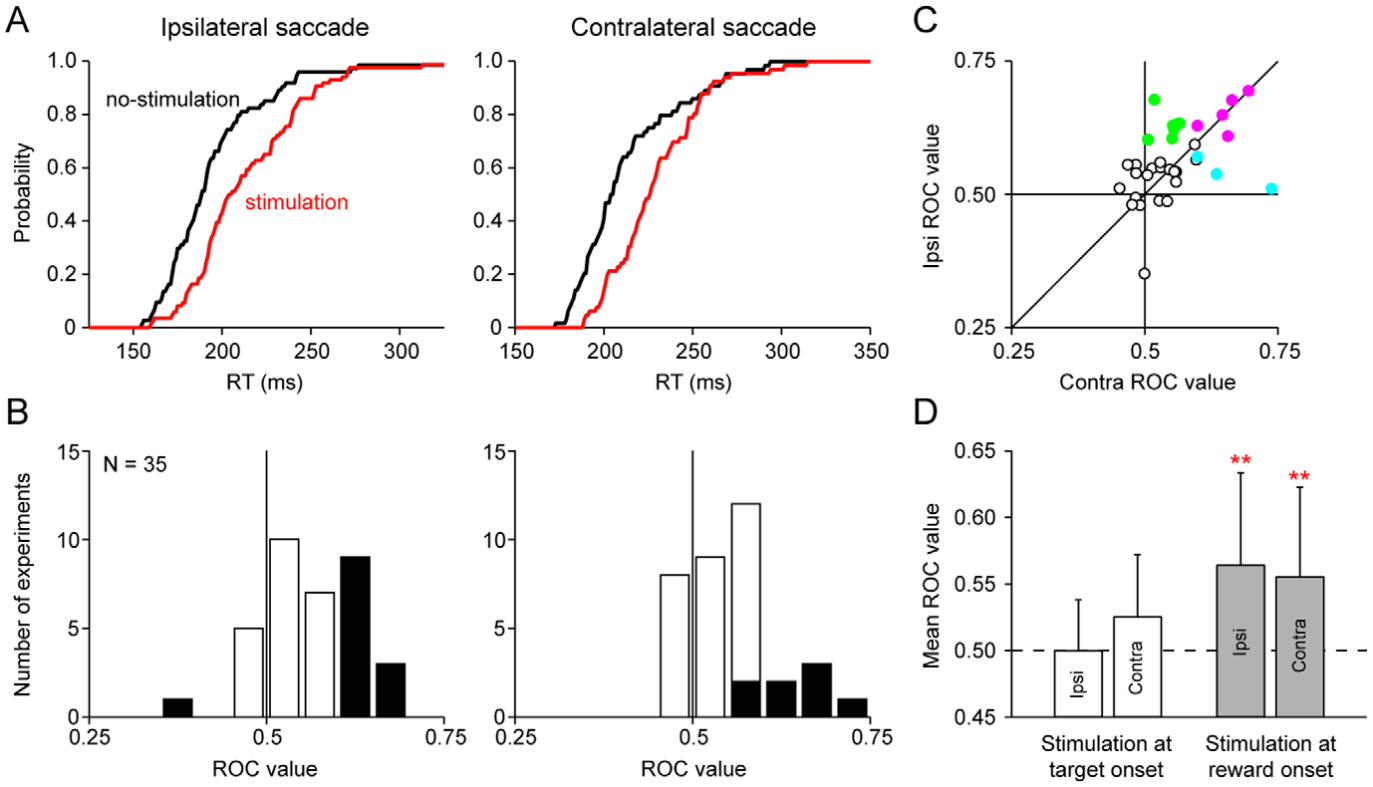
Effect of LHb stimulation on saccade latency. (*A*) Cumulative distribution of saccade latencies for ipsilateral saccades (left) and contralateral saccades (right). Red line indicates the cumulative distribution when saccades were followed by LHb stimulation (that is, stimulation condition). Black line indicates the cumulative distribution when saccades were not followed by LHb stimulation (that is, no-stimulation condition). (*B*) Distribution of ROC values comparing saccade latency between the stimulation and no-stimulation conditions for ipsilateral saccades (left) and contralateral saccades (right) (N = 35). ROC values more than 0.5 indicate longer latencies in the stimulation condition. Black bars indicate experiments with a significant difference in saccade latency between the stimulation and no-stimulation conditions (P<0.05, Wilcoxon rank-sum test). (*C*) Comparison of the ROC values between ipsilateral saccades (ordinate) and contralateral saccades (abscissa) (N = 35). Green, cyan and red dots indicate experiments with a significantly longer latency in the stimulation condition for ipsilateral saccades, contralateral saccades and both of them, respectively (P<0.05, Wilcoxon rank-sum test). White dots, no significance. (*D*) Mean ROC values in the original procedure (LHb stimulation at reward onset, gray bars, N = 35) and in the control procedure (LHb stimulation at target onset, white bars, N = 9). Double asterisks indicate significant deviation from 0.5 (P<0.01, Wilcoxon signed-rank test). Error bars indicate s.d.

We found that saccade latency increased when the saccade was followed by LHb stimulation. Figure 2*A* shows the cumulative distribution of saccade latencies in an example stimulation experiment. The mean latency was significantly longer in the stimulation condition than in the no-stimulation condition for both ipsilateral and contralateral saccades (P<0.01, Wilcoxon rank-sum test).

The same effect was observed in many stimulation sessions. Of the 35 stimulation experiments, many showed a significant increase in saccade latency in the stimulation condition (ipsilateral saccade, N = 12, mean latency  = 225 ms in the stimulation condition and 214 ms in the no-stimulation condition; contralateral saccade, N = 8, mean latency  = 227 ms in the stimulation condition and 216 ms in the no-stimulation condition; P<0.05, Wilcoxon rank-sum test). Only one experiment showed a significant decrease in the latency (ipsilateral saccade, N = 1; contralateral saccade, N = 0). To evaluate the stimulation effect, we calculated ROC value comparing saccade latency between the stimulation and no-stimulation conditions for each experiment (Fig. 2*B*). The mean ROC value was significantly larger than 0.5 for both ipsilateral and contralateral saccades (P<0.01, Wilcoxon signed-rank test), indicating that saccade latency was longer in the stimulation condition than in the no-stimulation condition. This indicates that the LHb stimulation delivered after a particular saccade suppressed the initiation of that saccade on future trials.

The stimulation effect was not biased toward the ipsilateral or contralateral direction. Figure 2*C* shows the comparison of the ROC value between ipsilateral and contralateral saccades. Although some experiments showed a significant delay by LHb stimulation in ipsilateral saccade only (green dots, N = 7) or contralateral saccade only (cyan dots, N = 3), there was no significant difference, on average, between ipsilateral and contralateral ROC values (P = 0.18, Wilcoxon signed-rank test).

### No direct effect of LHb stimulation on saccadic motor circuits

We so far found that the post-saccadic LHb stimulation suppressed the saccades on later trials. Because the stimulation period did not overlap with the preparation or execution time of the saccades, it is unlikely that the stimulation effect was caused by a direct influence on a saccadic motor mechanism. To exclude this possibility more clearly, we changed the timing of the LHb stimulation such that it started simultaneously with the target onset. This stimulation was operative during the preparation and execution of saccade.

We performed 9 stimulation experiments in one of the two monkeys using the same visually guided saccade task with the earlier stimulation timing. None of the experiments showed a significant increase or decrease in saccade latency by the stimulation (ipsilateral saccade, mean latency  = 233 ms in the stimulation condition and 233 ms in the no-stimulation condition; contralateral saccade, mean latency  = 214 ms in the stimulation condition and 212 ms in the no-stimulation condition; P>0.05, Wilcoxon rank-sum test). The average of the ROC values did not show a significant deviation from 0.5 (white bars in Fig. 2*D*) (P>0.05, Wilcoxon signed-rank test). These results suggest that the LHb has no direct control over the neural mechanisms underlying the preparation or execution of saccades.

### Gradual changes in saccade latency by post-saccadic LHb stimulation

**Figure 3:**
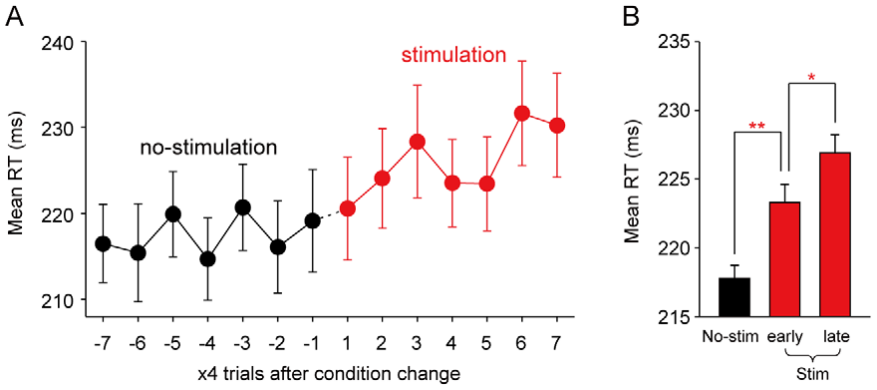
Time course of the change in saccade latency. (*A*) Mean saccade latencies plotted against the number of trials before and after direction-stimulation contingency was reversed. Red plots indicate mean latencies in the stimulation condition. Black plots indicate mean latencies in the no-stimulation condition. Each plot indicates the mean of latencies in 4 trials. (*B*) Mean saccade latency. Trials of the stimulation condition were divided into the early period (the first half of the 36 trials of the condition) and the late period (the latter half of the 36 trials), and the mean saccade latency was calculated for each period. Double and single asterisks indicate a significant difference (P<0.01 and P<0.05 respectively, Wilcoxon rank-sum test). Data from monkeys E and D as well as from ipsilateral and contralateral saccades were combined. Experiments with a significantly longer latency in the stimulation condition comprised the sample used for the analysis (ipsilateral saccades, N = 12; contralateral saccades, N = 8). Error bars indicate s.e.m.

If the LHb does not directly control saccadic motor circuits as suggested above, how did the post-saccadic LHb stimulation suppress the following saccades? The time course of the changes in saccade latency provides a useful suggestion. Figure 3*A* shows the mean saccade latency plotted against the number of trials after the direction-stimulation contingency was reversed. After the change from the no-stimulation condition (black plots) to the stimulation condition (red plots), saccade latency increased gradually as the saccade was repeatedly followed by LHb stimulation.

To quantify the gradual increase of saccade latency, we divided trials of the stimulation condition into the early period (the first half of the 36 trials after the change from the no-stimulation to the stimulation conditions) and the late period (the latter half of the 36 trials), and compared the mean saccade latency between the periods. As shown in Fig. 3*B*, the latency was significantly longer in the late period than in the early period (P<0.05, Wilcoxon rank-sum test), indicating that the increase of saccade latency was more robust in the late period.

The gradual change in saccade latency suggests that the effect of the post-saccadic LHb stimulation was accumulated across trials, and that the effect was mediated by a learning mechanism, rather than a motor execution mechanism.

### No effect of electrical stimulation of a neighboring structure on saccade latency

**Figure 4:**
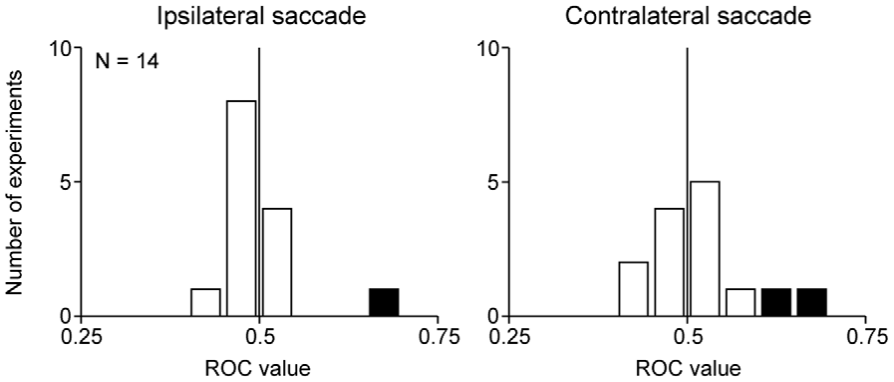
Effect of MD stimulation on saccade latency. Distributions of the ROC values comparing saccade latency between the stimulation and no-stimulation conditions are shown for ipsilateral saccades (left) and contralateral saccades (right) (N = 14). Conventions are the same as Fig. 2*B*.

To test whether the effect of the post-saccadic stimulation on saccade latency was actually caused by the LHb, we stimulated a neighboring structure, the mediodorsal nucleus of the thalamus (MD). We performed 14 stimulation experiments in the two monkeys using the same task procedure shown in Fig. 1 (including stimulation timing and stimulation parameters). However, only a few experiments showed a significant increase in saccade latency by the MD stimulation (ipsilateral saccade, N = 1; contralateral saccade, N = 2; P<0.05, Wilcoxon rank-sum test). We also calculated the ROC value comparing saccade latency between the stimulation and no-stimulation conditions (Fig. 4). The mean ROC value did not significantly deviate from 0.5 for both ipsilateral and contralateral saccades (P>0.05, Wilcoxon signed-rank test), indicating that saccade latency was not consistently affected by the MD stimulation. These results suggest that the effective stimulation sites were localized within the LHb.

## Discussion

It has been shown consistently that the initiation of a saccade is delayed if the saccade is followed by no reward (i.e., reward omission) [25]. We previously found that LHb neurons are activated phasically by unpleasant events including reward omission [18], [19]. We thus hypothesized that the phasic activation of LHb neurons is a key mechanism underlying the suppression of saccades caused by reward omission. The results of the present stimulation experiment support this hypothesis: LHb neurons were activated phasically by electrical stimulation (not unpleasant events) and consequently saccades were delayed.

Notably, the suppression (i.e., delay) of a saccade occurred gradually as the particular saccade was followed by LHb stimulation repeatedly. This gradual increase in saccade latency suggests that the effect of LHb stimulation was mediated by a learning mechanism, rather than a motor mechanism.

To test this ‘learning’ hypothesis more clearly, we also stimulated the LHb during the preparation and execution of saccades. Consistent with the hypothesis, we found no change in saccade latency, suggesting that the effect of LHb stimulation was not mediated by a motor mechanism underlying the preparation or execution of saccades. Notably, this stimulation, which started at the onset of saccadic target, overlapped not only with the preparation and execution periods of saccades but also with a part of the post-saccadic period because the stimulation (duration  = 800 ms) continued even after saccade onset (mean latency  = 225 ms) but not until reward onset (see method section for details). Therefore, effective LHb stimulation needs to be operative during reward delivery which would drive learning process.

Recent studies from other groups also showed the effect of electrical stimulation of the LHb on animal behavior. Friedman et al. (2010) reported that the deep brain stimulation (DBS) of the lateral habenula reduced cocaine seeking behavior in rats, suggesting that the LHb DBS attenuated the reinforcing effect of cocaine [26]. Shumake et al. (2010) reported that LHb stimulation disrupts reinforcement learning using a two-way active avoidance task in gerbils [27]. They stimulated the LHb briefly when the animal correctly avoided an aversive foot shock. The LHb stimulation resulted in an impairment of avoidance acquisition, suggesting that the stimulation blocked learning from correct avoidance. Together with our results, these studies indicate that the LHb can modulate both positive and negative reinforcement learning.

A candidate that mediates the effect of the LHb stimulation may be midbrain dopamine neurons. These neurons are inhibited by LHb stimulation [19], [28], [29], and excited by reward and inhibited by reward omission [30]. Recent studies from our laboratory have suggested that the reward-modulated activity of dopamine neurons plays a key role in the motivational control of saccadic eye movements [25], [31], [32]. These studies proposed that the efficacy of cortico-caudate synapses carrying visuo-saccadic signals is enhanced or depressed depending on the concurrent increase or decrease in dopaminergic inputs. More specifically, the LHb-induced inhibition of dopamine neuron activity after a saccade would attenuate the D2-mediated long-term depression on the cortico-caudate synapse mediating information on the saccade and, via the indirect pathway, lead to an enhanced inhibitory output of the basal ganglia [33]. Such a plastic effect would be accumulated by repeating LHb stimulation and consequently the initiation of the saccade would be suppressed.

Other studies also suggested the involvement of dopamine neurons in learning from negative feedback. It was proposed that elevated and reduced dopamine signals have opposite effects on D1 and D2 receptors [20], which are largely segregated in the direct and indirect pathway in the cortico-striatal loop [34]. Thus, the elevated dopamine signal activates the direct D1-mediated pathway (‘Go’ pathway) and deactivates the indirect D2-mediated pathway (‘NoGo’ pathway), driving learning to facilitate actions which lead to positive outcomes. Conversely, the reduced dopamine signal has the opposite effect, driving learning to avoid or suppress actions which lead to negative outcomes. Consistent with their theory, Klein et al. (2007) found that A1-allele carriers with reduced dopamine D2 receptor densities learned less efficiently to avoid actions leading to negative outcome [21]. These results are consistent with our hypothesis that the activity of LHb neurons, by inhibiting dopamine neuron activity, contributes to learning to suppress actions which lead to unpleasant events.

Another candidate mediating the effect of LHb stimulation may be serotonin neurons in the raphe nuclei. These neurons are also inhibited by LHb stimulation [35] and have been implicated in learning [36], [37]. The activity of neurons in the dorsal raphe nucleus, a major source of serotonin, is modulated by reward outcome [38] and the lack of serotonin in the prefrontal cortex impairs flexible learning to obtain rewards [39]. Future studies are called for to determine which route mediates the suppression effect of LHb stimulation on saccadic eye movements.

It has been shown repeatedly that rats with habenula lesions have difficulty in avoiding punishments [1], [16], [17]. Our studies on trained monkeys provide a neurophysiological account of avoidance behavior: An aversive stimulus or its predictor excites LHb neurons [18] and this LHb activation leads to the suppression of a motor behavior that is associated with the aversive stimulus (present study).
